# Selective ring-opening metathesis polymerization (ROMP) of cyclobutenes. Unsymmetrical ladderphane containing polycyclobutene and polynorbornene strands

**DOI:** 10.3762/bjoc.15.4

**Published:** 2019-01-03

**Authors:** Yuan-Zhen Ke, Shou-Ling Huang, Guoqiao Lai, Tien-Yau Luh

**Affiliations:** 1 Department of Chemistry, National Taiwan University, Taipei 106, Taiwan; 2 Shanghai Institute of Organic Chemistry, Chinese Academy of Sciences, 345 Lingling Lu, Shanghai 200032, China; 3 Key Laboratory of Organosilicon Chemistry and Material Technology of Ministry of Education, Hangzhou Normal University, Hangzhou, Zhejiang 311121, China

**Keywords:** cyclobutene, hydrolysis, linker, metathesis, norbornene, ROMP, selectivity, unsymmetrical ladderphane

## Abstract

At 0 °C in THF in the presence of Grubbs first generation catalyst, cyclobutene derivatives undergo ROMP readily, whereas norbornene derivatives remain intact. When the substrate contains both cyclobutene and norbornene moieties, the conditions using THF as the solvent at 0 °C offer a useful protocol for the selective ROMP of cyclobutene to give norbornene-appended polycyclobutene. Unsymmetrical ladderphane having polycyclobutene and polynorbornene as two strands is obtained by further ROMP of the norbornene appended polycyclobutene in the presence of Grubbs first generation catalyst in DCM at ambient temperature. Methanolysis of this unsymmetrical ladderphane gives polycyclobutene methyl ester and insoluble polynorbornene-amide-alcohol. The latter is converted into the corresponding soluble acetate. Both polymers are well characterized by spectroscopic means. No norbornene moiety is found to be incorporated into polycyclobutene strand at all. The double bonds in the polycyclobutene strand are mainly in *cis* configuration (ca 70%), whereas the *E*/*Z* ratio for polynorbornene strand is 8:1.

## Introduction

Ring-opening metathesis polymerizations (ROMP) of strained cycloalkenes offer a powerful arsenal for the synthesis of polymers having a variety of fascinating properties [1–3]. To illustrate this, polynorbornenes and polycyclobutenes are readily obtained from the corresponding monomeric norbornene and cyclobutene derivatives under various conditions. Symmetrical DNA-like double stranded ladderphanes are conveniently synthesized from bisnorbornene [4–15] or from biscyclobutene [16] linked with a range of different rigid linkers. When a flexible linker is used, bisnorbornene derivatives undergo cascade metathetical cyclopolymerization giving the corresponding polynorbornenes with hammock-like pendants [17–18]. Unsymmetrical polynorbornene-based ladderphane is obtained by a replication protocol from a single stranded polynorbornene [19–20]. Alternatively, sequential polymerization of a monomer containing a norbornene moiety and other polymerizable group furnishes an unsymmetrical ladderphane having two structurally different polymeric backbones [21–22]. It seems to be not easy if both strands are arisen from different strained rings by ROMP. It is known that norbornenes having different substituents would have different reaction rates in ROMP [23]. These discrepancies in reactivity have been used for sequence control in polymer synthesis [24]. Since the first living ROMP methods for cyclobutenes were reported in 1992 [25], cyclobutene-containing block copolymers are well documented [26–34]. Alternating cyclobutene–cyclohexene copolymers have been synthesized by ROMP of the corresponding monomers [31–33]. However, to the best of our knowledge, selective ROMPs between cyclobutene and norbornene have not been reported.

**Scheme 1:**
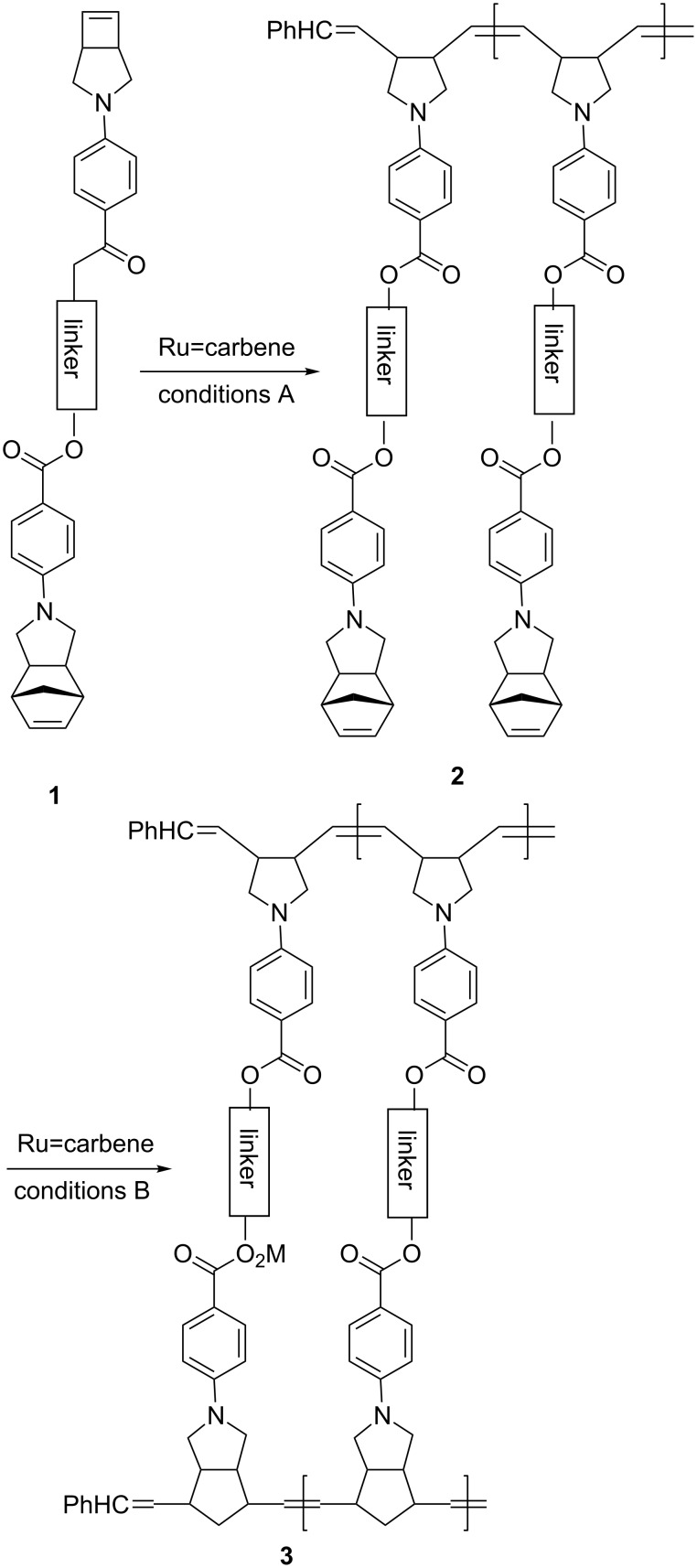
Strategy for sequential ROMP of **1** to yield **3**.

The strain energies for norbornene and cyclobutene are 25 and 31 kcal/mol, respectively [35]. It is therefore envisaged that cyclobutene would react faster than norbornene under certain ROMP conditions. As such, when monomer **1** containing a cyclobutene moiety and a norbornene moiety connected by a bridge are subjected to ROMP, it would be feasible that the cyclobutene moiety would react preferentially giving the corresponding norbornene-appended polycyclobutene **2**. After all cyclobutene moieties have been consumed and quenched, further ROMP of **2** under different conditions would afford unsymmetrical double-stranded ladderphane **3** having both polycyclobutene and polynorbornene as two polymeric frameworks (Scheme 1). We have tested this viewpoint and now wish to report sequential ROMP of monomers containing both cyclobutene and norbornene moieties tethered by a linker.

## Results and Discussion

### A comparison of the reactivity of cyclobutene versus norbornene derivatives **4** and **5** in ROMP catalyzed by Grubbs I catalyst (**6**)

In the beginning of this study, we have examined the first order reaction kinetics of ROMPs of **4** and of **5** in the presence of 10 mol per cent of Grubbs first generation catalyst (**6**) [36] in DCM at 10 °C [37]. The rate constants for the reactions of **4** and **5** were 1.3 × 10^−3^ and 5.1 × 10^−4^ s^−1^, respectively. On the other hand, when the reaction was carried out in THF-*d*_8_ at 273 K, the second order rate constant for **4** was 2.1 × 10^−3^ M^−1^s^−1^, whereas norbornene derivative **5** was inert under these conditions. The details are described in the Experimental section and Supporting Information File 1 (Figures S1, S2 and S8–S10).

**Scheme 2:**
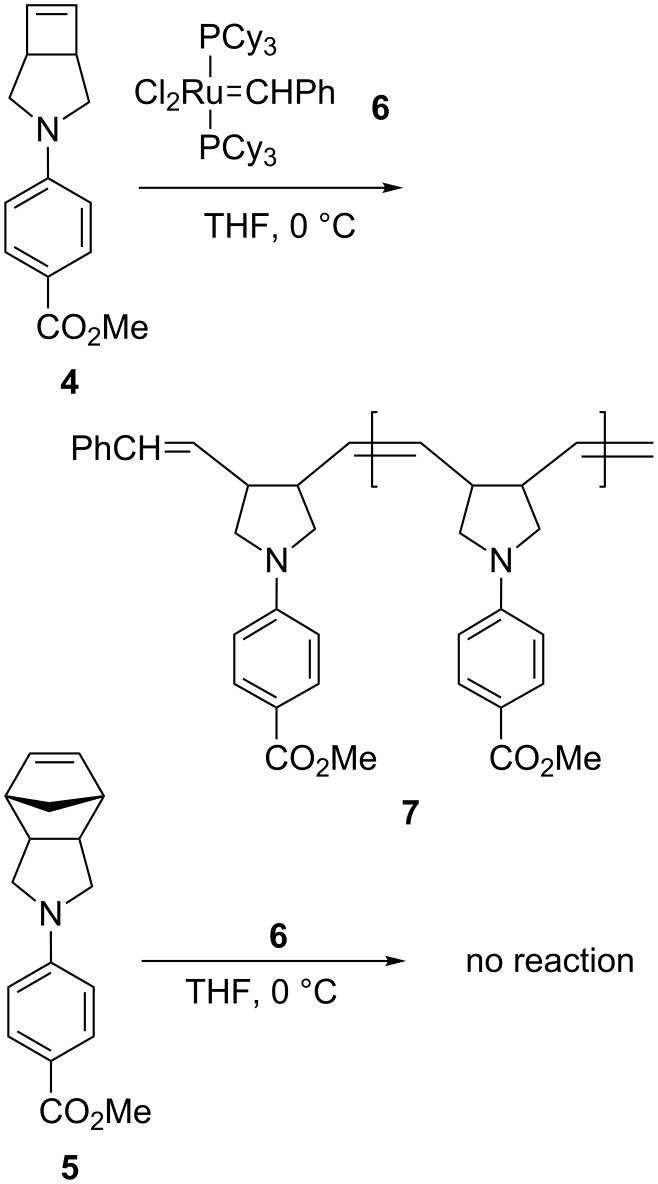
ROMP of **4** and **5** in THF at 0 °C in the presence of 10 mol % of **6**.

**Scheme 3:**
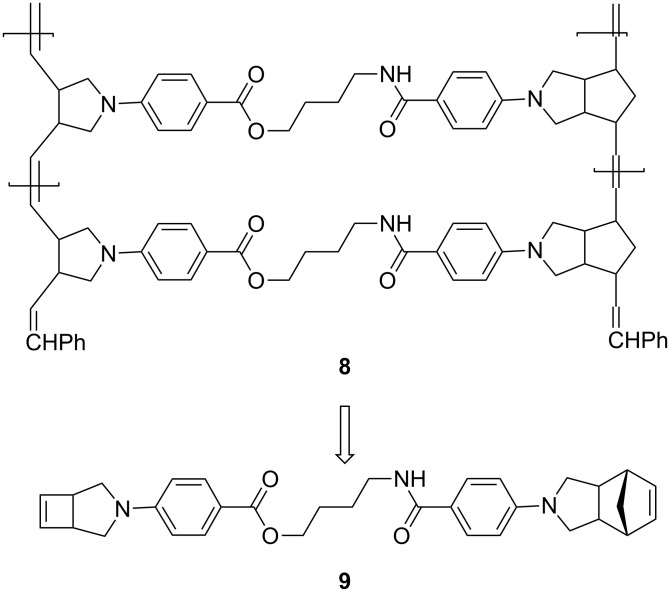
Retrosynthesis of **8** from **9**.

It has been suggested that the metathesis reaction may involve a fourteen-electron ruthenium species as the active catalyst [38–40]. This active species might be stabilized when the reaction is carried out in polar solvent having weak coordination ability such as THF [41–43]. As mentioned above, the difference in reactivity between the ROMP of **4** and **5** in THF at 0 °C would offer useful conditions to selectively react with **4** in the presence of **5**. Thus, a mixture of an equal molar of **4** and **5** was treated with 10 mol % of **6** in THF-*d*_8_ at 0 °C. Only **4** was consumed to give the corresponding polymer **7**, whereas **5** remained intact (Scheme 2). This promising observation prompted us to pursue the synthesis of unsymmetrical double-stranded ladderphane **8** by sequential ROMPs of **9** (Scheme 3).

### Synthesis of monomer **9**

**Scheme 4:**
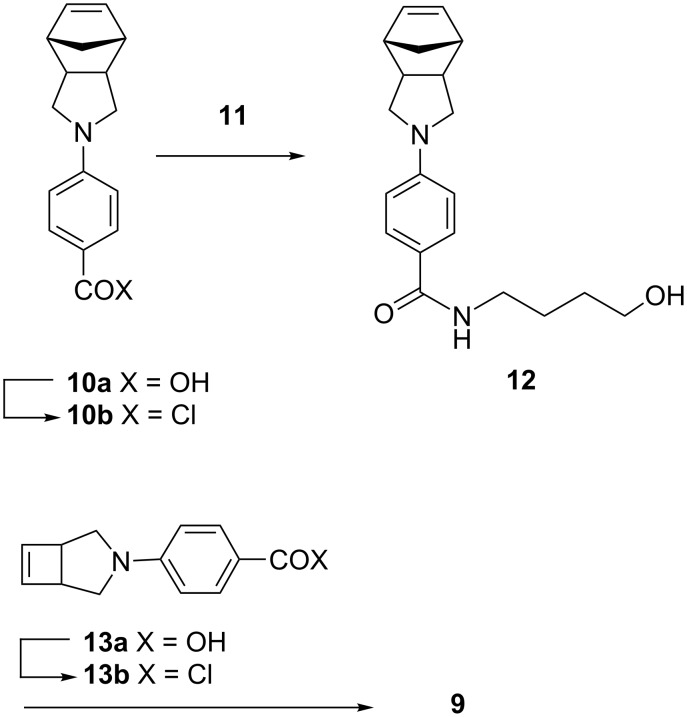
Synthesis of monomer **9**.

4-Aminobutanol (**11**) was used to link norbornene and cyclobutene moieties via amide and ester groups. The use of such a linker is because the ester group could be selectively hydrolyzed in the presence of amides. This selectivity will be helpful for the structural elucidation of polymer **8**. Thus, **10b** was allowed to react with **11** to afford amide-alcohol **12** in 79% yield. Esterification of **12** with **13b** furnished 70% yield of monomer **9** (Scheme 4).

### Synthesis of unsymmetrical ladderphane **8** by sequential ROMPs catalyzed by **6**

**Scheme 5:**
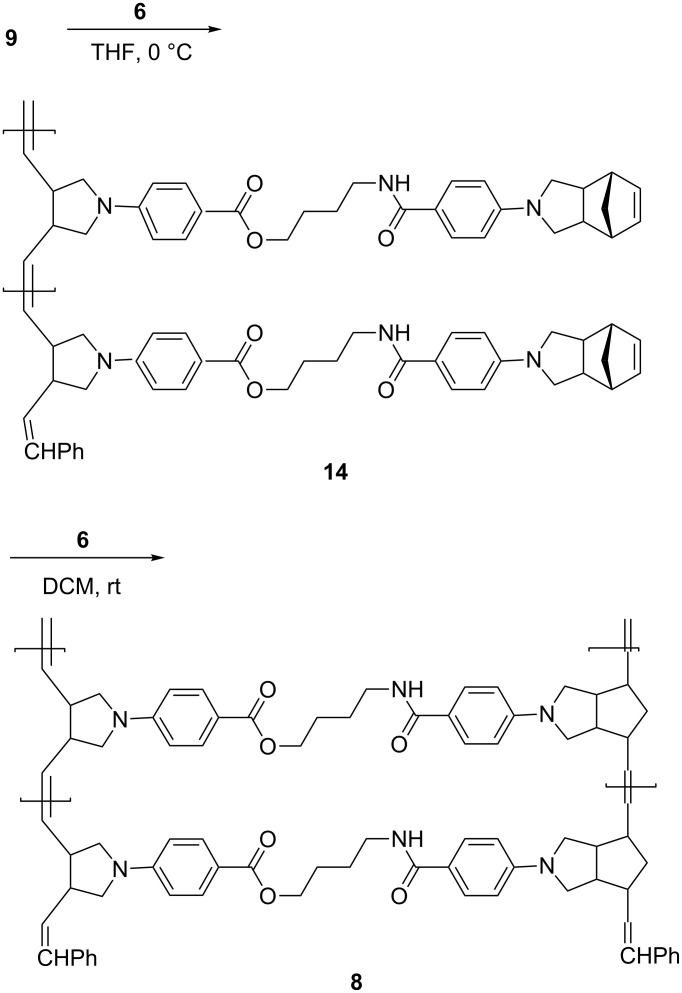
Synthesis of **14** and **8** by selective olefin metathesis.

Polymerization of monomer **9** in the presence of 10 mol % of **6** was performed in THF at 0 °C for 4 h, followed by quenching with ethyl vinyl ether to give polymer **14** in 86% yield (Scheme 5). It is worth noting that no incorporation of the norbornene moiety into the polymeric backbone under these conditions was observed. The ^1^H NMR spectrum of **14** shows the olefinic proton signals at δ 5.49 and 6.12 ppm in 1:1 ratio. These signals were assigned to the absorptions of olefinic protons on the polymeric backbone and the olefinic proton of unreacted norbornene pendants, respectively. In the ^13^C NMR spectrum, the peak at δ 139 ppm owing to the olefinic carbon of cyclobutene shifts to δ 130 ppm due to ring opening, whereas the olefin carbon of the unreacted norbornene moiety at δ 136 ppm remained unchanged after first polymerization. These observations are consistent with the results of our preliminary studies that only the cyclobutene moiety, but not norbornene in **9**, proceeds **6**-catalyzed ROMP under these conditions. The degree of polymerization of **14** was estimated to be 10 based on the ^1^H NMR integration of relevant peaks.

**Scheme 6:**
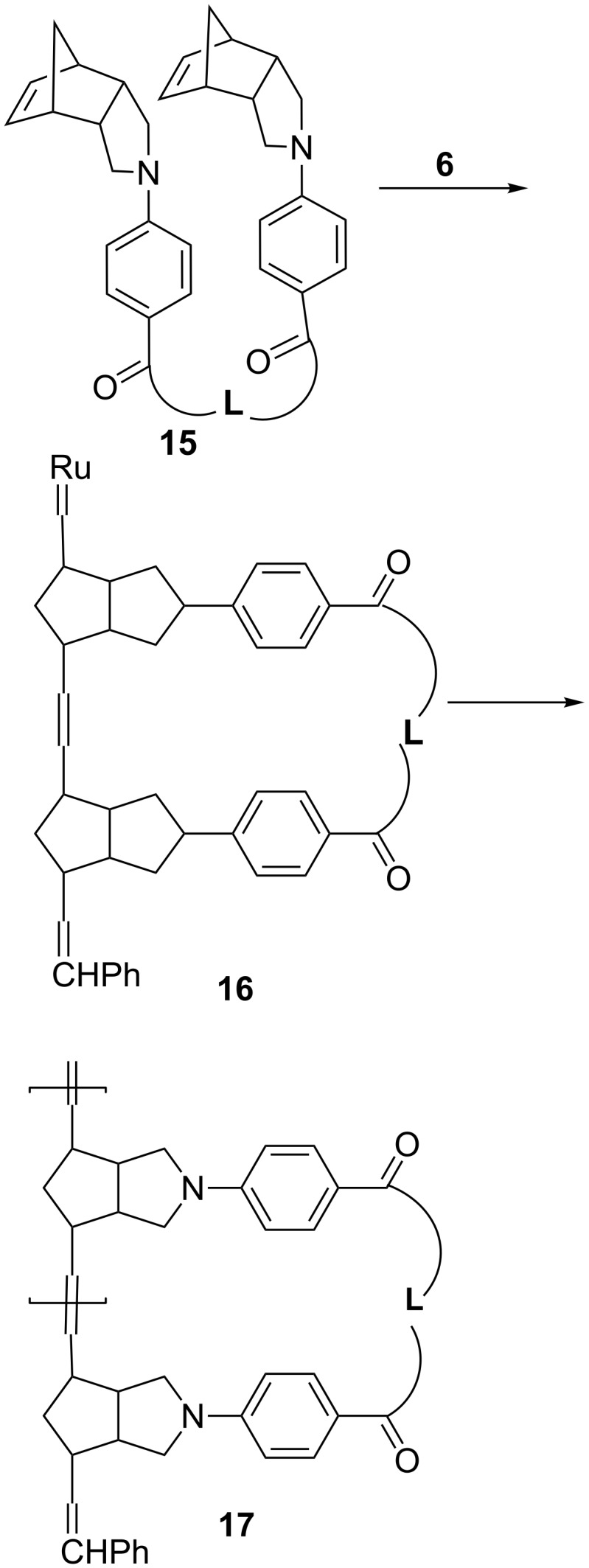
Cyclopolymerization of **15** with a flexible linker.

We have previously found that two norbornene derivatives connected by a flexible linker **15** may undergo cascade ring-opening–ring-closing metathesis polymerization to give single-stranded hammock-like appended polynorbornenes **17** (Scheme 6) [17–18]. The linker in **8** is flexible, and, therefore, the possibility for similar intramolecular metathesis cyclopolymerization might take place to form intermediate **16** for further transformations. However, no such reaction was observed in this study. Presumably, the **6**-catalyzed metathesis reactivity of cyclobutenes would be much higher than that of norbornene derivatives. Accordingly, intermolecular metathesis reaction between two cyclobutene moieties would be favored over intramolecular ring-closing metathesis between a ruthenium carbene and the norbornene moiety.

Polymer **14** was treated with 10 mol % **6** in DCM at rt to give **8** in 95% yield. The ^1^H NMR spectrum of **8** shows that the relative intensity of the signals around δ 5.4 ppm was doubled, all signals due to olefinic protons in **9** and **14** being diminished.

### Methanolysis of unsymmetrical ladderphane **8**

**Scheme 7:**
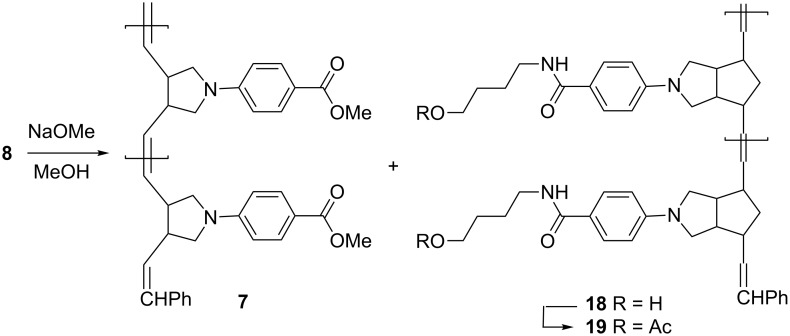
Methanolysis of unsymmetrical ladderphane **8**.

In order to confirm the uniformity of the polymerization leading to the formation of unsymmetrical ladderphane **8**, methanolysis of **8** with NaOMe in methanol at rt gave **7** and **18**. Chloroform was then added and **18** was collected as a grayish precipitate in 56% yield. After filtration, the filtrate was worked up to afford **7** in 64% yield with a degree of polymerization of 10 (*M*_n_ = 2500, PDI = 1.11), in good agreement with those of **14** and **8**. The ^13^C NMR spectrum of **7** shows two peaks at δ 40.6 and 45.4 ppm, attributed to the allylic carbons attached to a *cis* and a *trans* double bond [13], respectively, and the relative ratio of these two peaks is roughly 7:3. This result suggests that about 70% of the double bonds in **7** might adopt *cis* configuration. Moreover, no norbornene moiety was detected by NMR on the polymeric backbones in **7** (Scheme 7).

Since **18** was insoluble in most organic solvents, acetylation of **18** with excess acetic anhydride and pyridine at 70 °C for 10 h gave the corresponding acetate **19**, which had good solubility in DCM or chloroform. GPC analysis showed that the degree of polymerization of **19** (DP = 10, PDI = 1.24) was again comparable with that of the corresponding ladderphane **8**, polycyclobutene **7** and **14**.

The ^1^H NMR spectrum of **19** shows peaks at δ 5.6 and 5.3 ppm attributed to *trans* and *cis* olefinic protons, respectively, in a ratio of 8 to 1. It is well documented that **6**-catalyzed ROMP of *N*-arylpyrrolidene appended norbornene gives polynorbornene with all double bonds in *trans* configuration [44–46]. The existence of both *Z*- and *E*-double bonds in the parent polycyclobutene backbone in **14** may influence the stereoselectivity of the polynorbornene strand in **7** during the course of ROMP.

## Conclusion

In summary, we have demonstrated useful ROMP conditions to selectively transform cyclobutene derivatives into the corresponding polycyclobutenes in THF at 0 °C, whereas the corresponding norbornene skeleton appears to be unreactive under these conditions. This protocol has been used for the selective synthesis of unsymmetrical ladderphane having polycyclobutene in one strand and polynorbornene in the other. Further applications of this selectivity to other systems are in progress in our laboratory.

## Experimental

### General

Unless otherwise specified, all commercially available starting materials were used without further purification. All air and moisture-sensitive reactions were carried out under an atmosphere of dry nitrogen in a glove box. All ^1^H and ^13^C NMR spectra were recorded on a Varian 400 Unity Plus NMR spectrometer using CDCl_3_ as solvent at ambient temperature. Chemical shifts were expressed in parts per million using residual solvent protons as internal standards (^1^H: chloroform: 7.26 ppm). Gel permeation chromatography (GPC) was performed on a Waters GPC instrument equipped with Waters 1515 HPLC pump using Waters 2487 absorbance detector. Polymer (approximately 0.5 mg) in THF (0.1 mL) was filtered through a 0.5-micron filter and 20 μL of the sample was injected into Shodex KF-G, Styragel HR2, Styragel HR3 and Styragel HR4 column (7.8 × 300 mm) with oven temperature at 40 °C using standard polystyrene samples (1.84 × 10^5^ to 996 Da) for calibration. THF was used as eluent (flow rate 1.0 mL/min).

**Synthesis of 12.** Under N_2_ atomosphere, to **10a** (560 mg, 2.2 mmol) in DCM (20 mL) was added oxalyl chloride (0.4 mL, 4.3 mmol) at 0 °C. The mixture was gradually warmed to rt and then stirred for 1 h. The solvent was removed in vacuo to give the crude acyl chloride **10b**, to which was added DCM (15 mL), DMAP (60 mg, 0.5 mmol) and Et_3_N (2.0 mL, 15 mmol). 4-Amino-1-butanol (**11**, 178 mg, 2.0 mmol) was then added slowly at 0 °C. After stirring for 8 h at rt, the mixture was poured into H_2_O (50 mL) and DCM (50 mL). The organic layer was separated, washed with brine (100 mL) and dried (MgSO_4_). The solvent was removed in vacuo and the residue was chromatographed on silica gel (DCM/MeOH 20:1) to afford **12** (515 mg, 79%). mp 207–209 °C; IR (KBr): ν 3455, 3306, 3056, 2940, 2867, 1606, 1554, 1514, 1473, 1379, 1309, 1199, 1130, 1047, 969, 826, 768, 733, 683 cm^−1^; ^1^H NMR (400 MHz) δ 1.52 (d, *J* = 8.4 Hz, 1H), 1.61–1.70 (m, 6H), 2.92–2.99 (m, 4H), 2.98–2.99 (m, 2H), 3.09 (m, 2H), 3.25–3.30 (m, 2H), 3.47–3.48 (m, 2H), 3.71 (t, *J* = 5.6 Hz, 2H), 6.15–6.16 (m, 3H), 6.39 (d, *J* = 9.0 Hz, 2H), 7.61 (d, *J* =9.0 Hz, 2H); ^13^C NMR (100 MHz): δ 26.8, 30.1, 39.8, 45.6, 46.8, 50.6, 52.2, 62.3, 110.7, 120.1, 127.9, 135.3, 148.9, 167.1; HRMS (FAB, *m*/*z*): calcd for C_20_H_26_N_2_O_2_, 326.1994; found, 326.1997.

**Synthesis of 9.** Under N_2_ atomosphere, to **13a** (321 mg, 1.4 mmol) in DCM (20 mL) was added oxalyl chloride (0.4 mL, 4.3 mmol) at 0 °C. The mixture was gradually warmed to rt and then stirred for 1 h. The solvent was removed in vacuo to give the crude acyl chloride **13b**, to which was added DCM (15 mL), DMAP (60 mg, 0.5 mmol) and Et_3_N (2.0 mL, 15 mmol). Compound **12** (522 mg, 1.6 mmol) was then added slowly at 0 °C. After stirring for 8 h at rt, the mixture was poured into H_2_O (50 mL) and DCM (50 mL). The organic layer was separated, washed with saturated brine (100 mL) and dried (MgSO_4_). The solvent was removed in vacuo and the residue was chromatographed on silica gel (DCM/MeOH 20:1) to afford **9** (512 mg, 70%). mp 238–240 °C; IR (KBr): ν 3333, 3051, 2949, 2843, 1699, 1606, 1547, 1511, 1473, 1376, 1274, 1216, 1180, 1106, 1050, 963, 828, 769, 740 cm^−1^; ^1^H NMR (400 MHz) δ 1.51 (d, *J* = 8.2 Hz, 1H), 1.61 (d, *J* = 8.2 Hz, 1H), 1.72–1.85 (m, 4H), 2.92–2.98 (m, 6H), 3.07–3.09 (m, 2H), 3.25–3.29 (m, 2H), 3.49–3.56 (m, 4H), 3.65 (d, *J* = 10.0 Hz, 2H), 4.30 (t, *J* = 6.4 Hz, 2H), 6.03 (m, 1H), 6.13–6.15 (m, 4H), 6.38 (d, *J* = 8.6 Hz, 2H), 6.62 (d, *J* = 8.4 Hz, 2H), 7.61 (d, *J* = 8.6 Hz, 2H), 7.87 (d, *J* = 8.4 Hz, 2H); ^13^C NMR (100 MHz) δ 26.4, 26.6, 39.4, 45.3, 46.4, 46.5, 48.8, 50.4, 52.0, 63.7, 110.8, 111.8, 117.5, 120.4, 128.0, 130.9, 135.5, 139.1, 149.1, 152.9, 166.6, 167.1; HRMS (FAB, *m*/*z*): calcd for C_33_H_37_N_3_O_3_, 523.2835; found, 523.2839.

**Synthesis of 14.** Under N_2_ atomosphere, to a solution of **9** (84.0 mg, 0.16 mmol) in THF (10 mL) was added **6** (12.8 mg, 0.016 mmol) in THF (1 mL) at 0 °C. After stirring at 0 °C for 4 h, ethyl vinyl ether (1.0 mL) was then added and stirring was continued at 0 °C for 2 h. The mixture was concentrated and the residual solution was added to methanol. The precipitate was collected and redissolved in DCM. Reprecipitation by adding the DCM solution to methanol afforded **14** as a grayish powder (74.8 mg, 89%). IR (KBr): ν 3350, 3054, 2954, 2847, 1695, 1605, 1512, 1476, 1381, 1275, 1179, 1107, 967, 827, 768, 733, 698 cm^−1^; ^1^H NMR (400 MHz) δ 1.51–1.72 (m, 6H), 2.92–3.48 (m, 16H), 4.26 (br, 2H), 5.49 (m, 2H), 6.12 (br, 2H), 6.36 (m, 5H), 7.63 (br, 2H), 7.86 (br, 2H); degree of polymerization (DP) analysis: δ 7.86/δ 5.07 = 10, indicating a DP of 10; ^13^C NMR (100 MHz) δ 26.6, 39.6, 40.9, 45.5, 46.6, 50.5, 52.1, 52.9, 64.0, 110.5, 110.9, 117.0, 120.5, 128.2, 129.8, 131.3, 135.6, 149.2, 150.2, 166.8, 167.4.

**Synthesis of 8.** Under N_2_ atomosphere, to a solution of **14** (62.8 mg, 0.12 mmol) in DCM (40 mL) was added **6** (9.6 mg, 0.012 mmol) in DCM (5 mL). After stirring at rt for 4 h, ethyl vinyl ether (0.5 mL) was then added and stirring was continued for 30 min. The mixture was concentrated and the residual solution was added to methanol. The precipitate was collected and redissolved in DCM. Reprecipitation by adding the DCM solution to methanol afforded **8** as a grayish powder (59.7 mg, 95%). IR (KBr): ν 3373, 3054, 2929, 2849, 1694, 1605, 1512, 1478, 1381, 1274, 1179, 1106, 966, 827, 767, 733, 697 cm^−1^; ^1^H NMR (400 MHz) δ 1.47 (br, 1H), 1.82 (m, 5H), 2.88–3.49 (m, 16H), 4.27 (br, 2H), 5.47 (m, 4H), 6.49 (m, 5H), 7.67–7.89 (m, 4H); DP analysis: δ 4.27/δ 5.05 = 11, indicating a DP of 11. ^13^C NMR (100MHz) δ 26.6, 40.0, 46.1, 49.7, 53.2, 63.7, 110.6, 111.8, 116.9, 121.8, 126.0, 128.5, 131.3, 136.5, 138.7, 150.1, 166.7, 167.5.

**Synthesis of 7 and 18.** To a solution of **8** (52 mg, 0.1 mmol [calculated based on the molecular weight of the monomeric unit]) in DCM (20 mL) was added 30% NaOMe in methanol (6 mL). The mixture was stirred at 50 °C for 20 h and cooled to rt. The insoluble solid residue was collected and dried to give crude **18** as a grayish solid (18 mg, 56%). After filtration, the filtrate was washed with water and dried (MgSO_4_). The mixture was concentrated and the residual solution was added to methanol. The precipitate was collected and redissolved in DCM. Reprecipitation by adding the DCM solution to methanol afforded **7** as a grayish powder (21 mg, 64%). IR (KBr): ν 3066, 2951, 2862, 1702, 1605, 1524, 1478, 1434, 1383, 1281, 1180, 1108, 970, 828, 769, 698, 507 cm^−1^; ^1^H NMR (400 MHz) δ 3.02–3.49 (m, 6H), 3.86 (br, 3H), 5.49 (m, 2H), 6.43 (br, 2H), 7.87 (br, 2H), DP analysis by integration of peaks at δ 6.43/δ 5.06 = 10, indicating a DP of 10. ^13^C NMR (100 MHz) δ 40.8, 45.8, 51.6, 52.7, 110.5, 117.1, 128.4, 129.7, 131.3, 150.2, 167.2. GPC: *M*_n_ = 2500, *M*_w_ = 2800 , PDI = 1.11.

**Synthesis of 19.** A mixture of crude **18** (16 mg, 0.05 mmol), obtained from the above experiment, in Ac_2_O (0.5 mL) and pyridine (5 mL) was stirred at 70 °C for 10 h. The solvent was concentrated and the residue was dissolved in CHCl_3_ (15 mL) and washed first with diluted HCl (pH 3) and then with water. The organic solvent was concentrated and the residual solution was added to methanol. The precipitate was collected and redissolved in CHCl_3_. Reprecipitation by adding the CHCl_3_ solution to methanol afforded **19** as a grayish powder (12 mg, 63%). ^1^H NMR (400 MHz) δ 1.73 (br, 6H) 2.05 (s, 3H), 2.73–3.62 (m, 10H), 4.07 (br, 2H), 5.50 (m, 2H), 6.48 (br, 2H), 7.73 (br, 2H), DP δ 5.50/δ 5.05 = 10, indicating a DP of 10. ^13^C NMR (100 MHz) δ 21.1, 28.0, 39.7, 45.0, 46.5, 50.8, 64.3, 112.2, 121.9, 128.5, 131.8, 132.0, 150.5, 168.1, 171.6.

### General procedure for kinetic measurements

Monomer **4** or **5** (0.03 mmol) was dissolved in DCM-*d*_2_ or THF-*d*_8_ (0.5 mL) and was syringed into an NMR tube inside a glove-box under nitrogen atmosphere. The NMR tube was then covered with a standard tube cap and placed in the NMR spectrometer. The tube was left to equilibrate at the desired temperature and all parameters were adjusted. A solution of **6** (24 mg in 1.0 mL of the same solvent) was prepared under nitrogen atmosphere prior to the reaction. Catalyst **6** (10 mol %) was syringed into the NMR tube which was immediately put in the NMR probe again. The reaction was monitored by the decrease of the peak intensity for H-2 using the peaks for H-1 and H-1’ as the internal reference (Supporting Information File 1, Figures S8–S10). The spectra were recorded every ten to twenty minutes interval depending on the reaction (Figures S8–S10). The rate constants were thus obtained (Figures S1 and S2).

## Supporting Information

File 1 ^1^H and ^13^C NMR spectra of both monomers and polymers, as well as GPC and kinetic investigation results.
