# Supplementary material for: Selective ring-opening metathesis polymerization (ROMP) of cyclobutenes. Unsymmetrical ladderphane containing polycyclobutene and polynorbornene strands
Source: Beilstein J Org Chem. 2019 Jan 3;15:44–51. doi: 10.3762/bjoc.15.4 (PMC6334803; doi:10.3762/bjoc.15.4)
Supplement: File 1 — 1H and 13C NMR spectra of both monomers and polymers, as well as GPC and kinetic investigation results. [file Beilstein_J_Org_Chem-15-44-s001.pdf]

BEILSTEIN JOURNAL OF ORGANIC CHEMISTRY

Supporting Information

for

Selective ring-opening metathesis polymerization (ROMP) of cyclobutenes. Unsymmetrical ladderphane containing polycyclobutene and polynorbornene strands

Yuan-Zhen Ke, Shou-Ling Huang, Guoqiao Lai and Tien-Yau Luh

Beilstein J. Org. Chem. **2019,** *15,* 44–51. doi:10.3762/bjoc.15.4

^{1}H and ^{13}C NMR spectra of both monomers and polymers, as well as GPC and kinetic investigation results

License and Terms: This is a supporting information file under the terms of the Creative Commons Attribution License (http://creativecommons.org/licenses/by/4.0). Please note that the reuse, redistribution and reproduction in particular requires that the authors and source are credited.
The license is subject to the *Beilstein Journal of Organic Chemistry* terms and conditions: (https://www.beilstein-journals.org/bjoc)

Figure S1: (a) Monomer conversion vs. time for **4** (solid square) and **5** (open circle) (0 °C, DCM-d_2); (b) First-order decay of **4** (solid square) and **5** (open circle) (0 °C, DCM-d_2).

Figure S2: (a) Monomer conversion vs. time for **4** (solid square) and **5** (open circle) (0 °C, THF-d_8); Right: Second-order decay of **4** (solid square) (0 °C, THF-d_8).

Figure S3: (a) ^{1}H (400 MHz) and (b) ^{13}C (100 MHz) NMR spectra of **9**..

Figure S4: (a) ^{1}H (400 MHz) and (b) ^{13}C (100 MHz) NMR spectra of **14**.

Figure S5: (a) ^{1}H (400 MHz) and (b) ^{13}C (100 MHz) NMR spectra of **8**.

Figure S6: (a) ^{1}H (400 MHz) and (b) ^{13}C (100 MHz) NMR spectra of **7**.

Figure S7: ^{1}H (400 MHz) and (b) ^{13}C (100 MHz) spectra of **19**.

Figure S8: Kinetics of the **6**-catalyzed reaction of **4** in CD_2Cl_2 at 10 ^{o}C The reaction was monitored by the decrease of the peak intensity for H-2 using the peaks for H-1 and H-1’ as the internal reference. The spectra were recorded every ten minutes.

Figure S9: Kinetics of the **6**-catalyzed reaction of **5** in CD_2Cl_2 at 10 ^{o}C The reaction was monitored by the decrease of the peak intensity for H-2 using the peaks for H-1 and H-1’ as the internal reference. The spectra were recorded every ten minutes.

Figure S10: Kinetics of the **6**-catalyzed reaction of **4** in THF at 0 ^{o}C The reaction was monitored by the decrease of the peak intensity for H-2 using the peaks for H-1 and H-1’ as the internal reference. The spectra were recorded every twenty minutes.

(a)

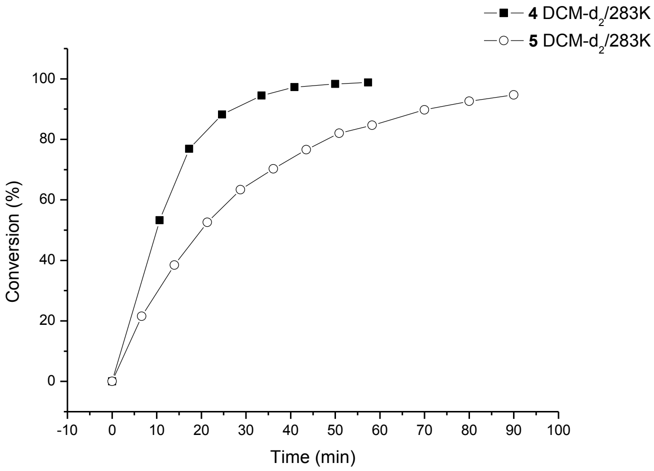

(b)

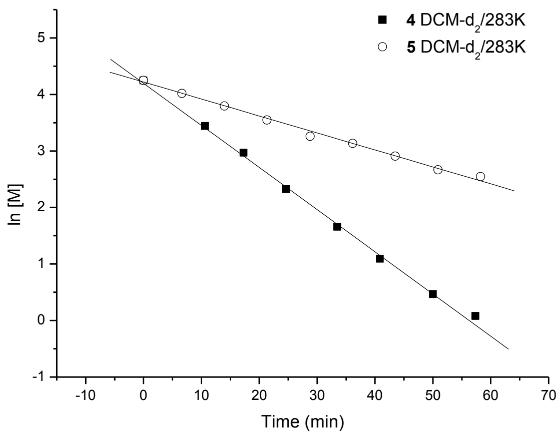

Figure S1: (a) Monomer conversion vs time for **4** (solid square) and **5** (open circle) (0 ^{o}C, DCM-d_2); (b) First-order decay of **4** (solid square) and **5** (open circle) (0 °C, DCM-d_2).

(a)

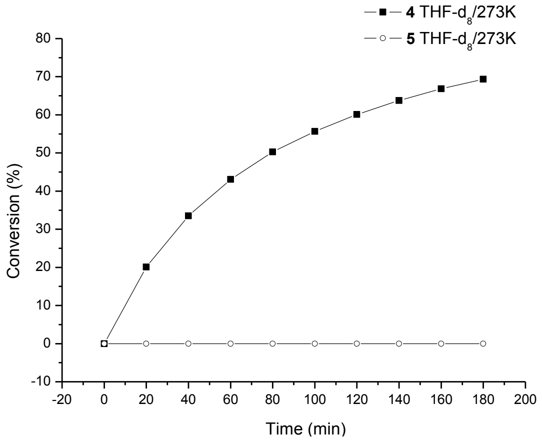

(b)

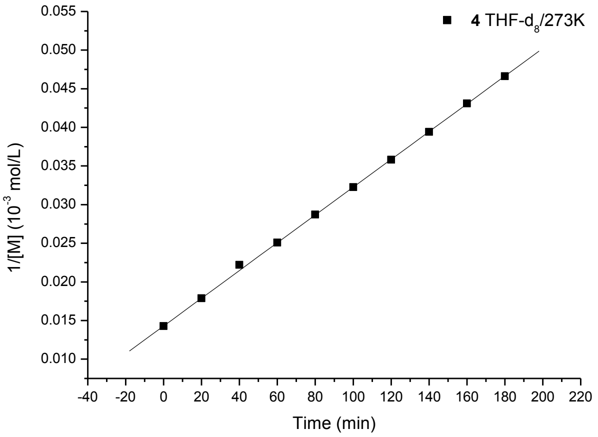

Figure S2: (a) Monomer conversion vs time for **4** (solid square) and **5** (open circle) (0 ^{o}C, THF-d_8); (b) Second-order decay of **4** (solid square) (0 °C, THF-d_8).

(a)

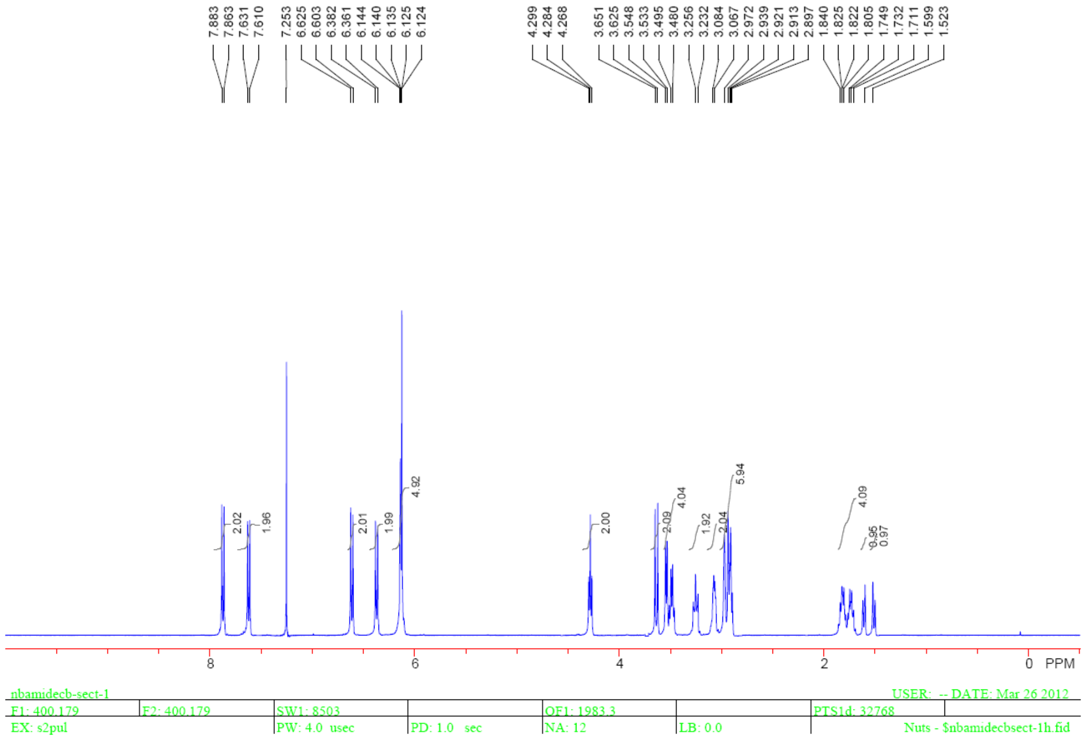

(b)

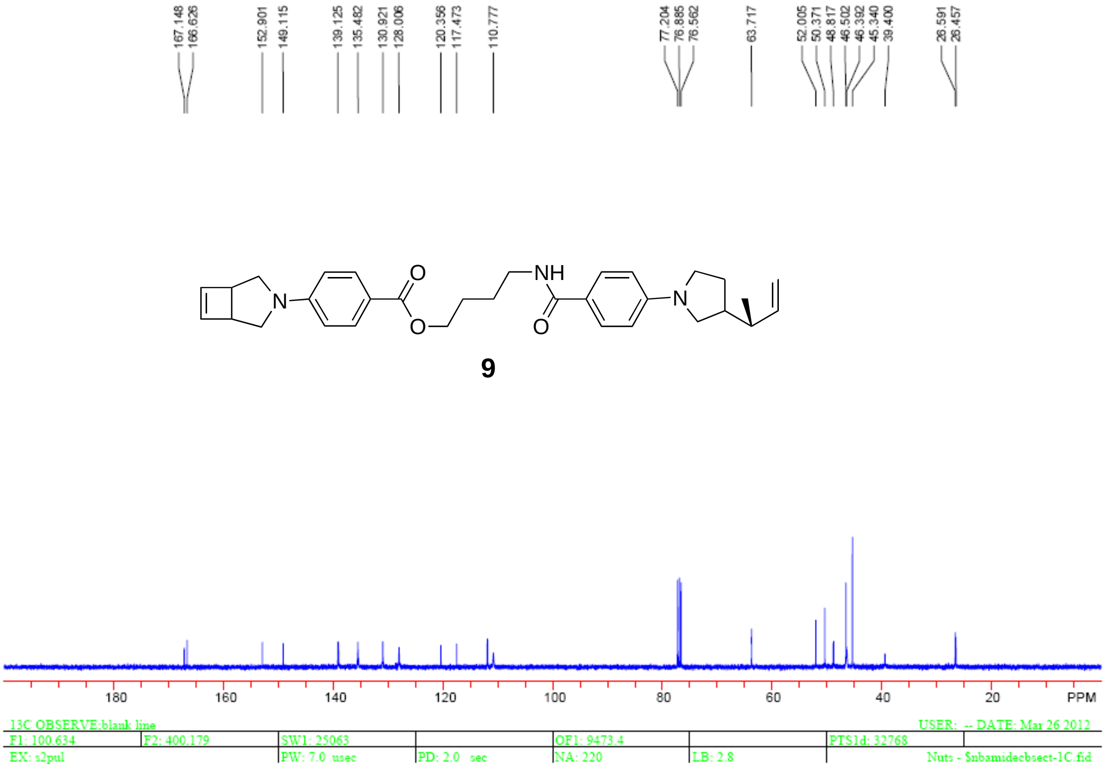

Figure S3: (a) ^{1}H (400 MHz) and (b) ^{13}C NMR (100 MHz) spectra of **9**.

(a)

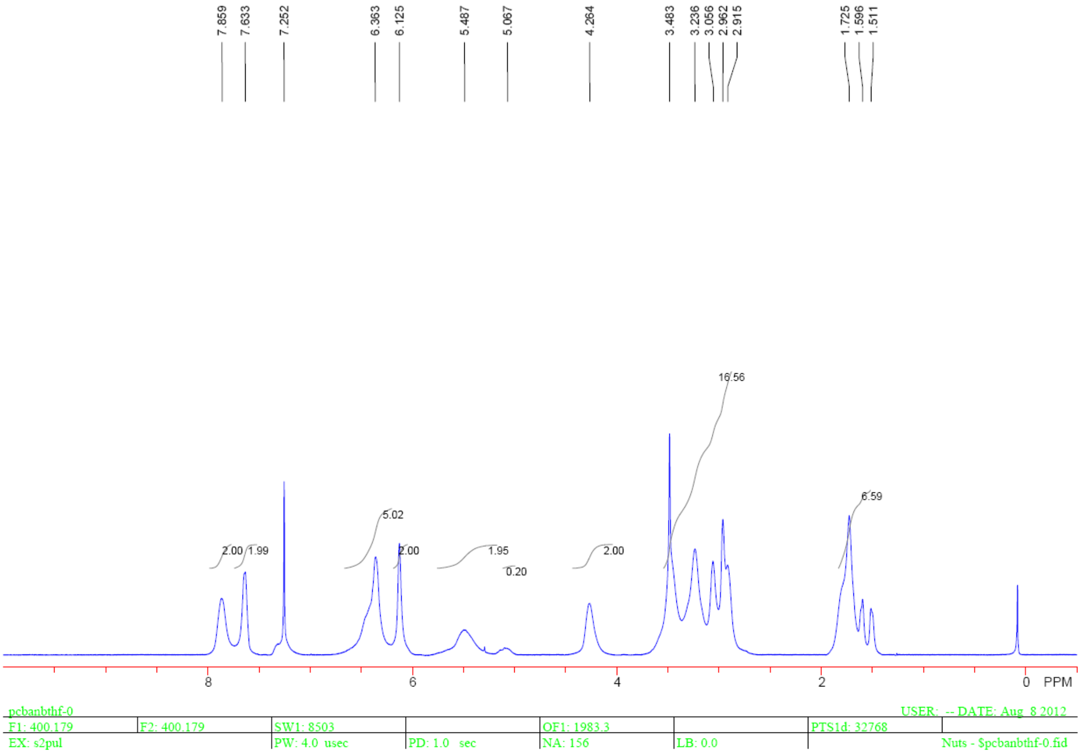

(b)

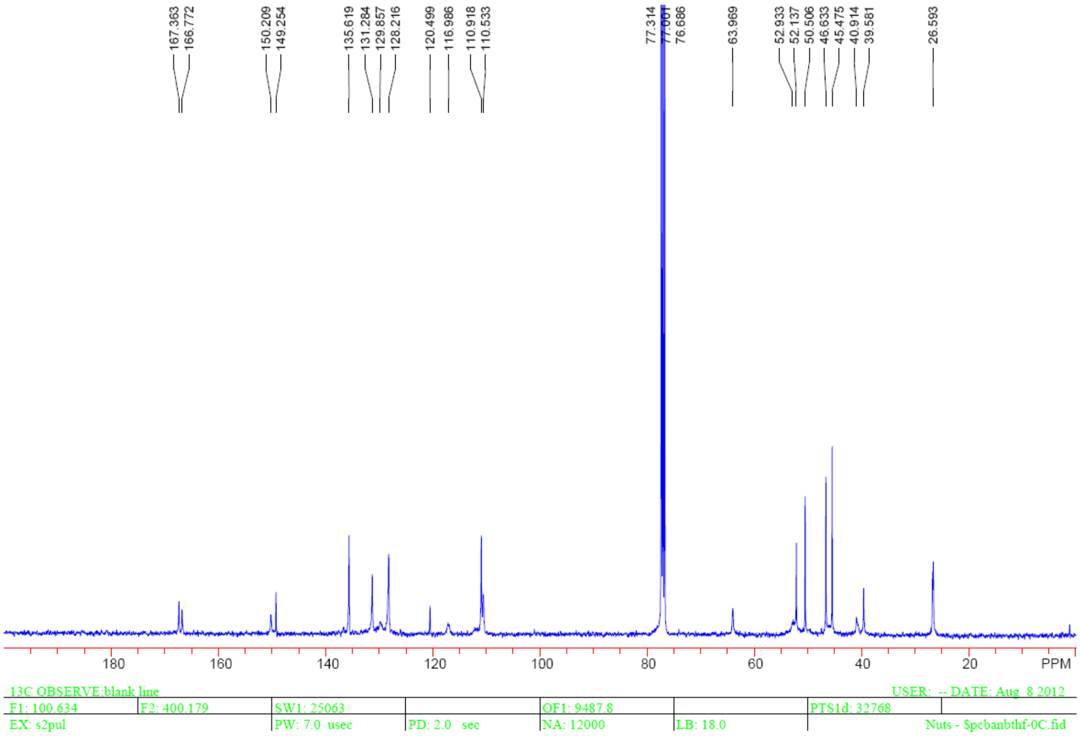

Figure S4: (a) ^{1}H (400 MHz) and (b) ^{13}C (100 MHz) NMR spectra of **14**.

(a)

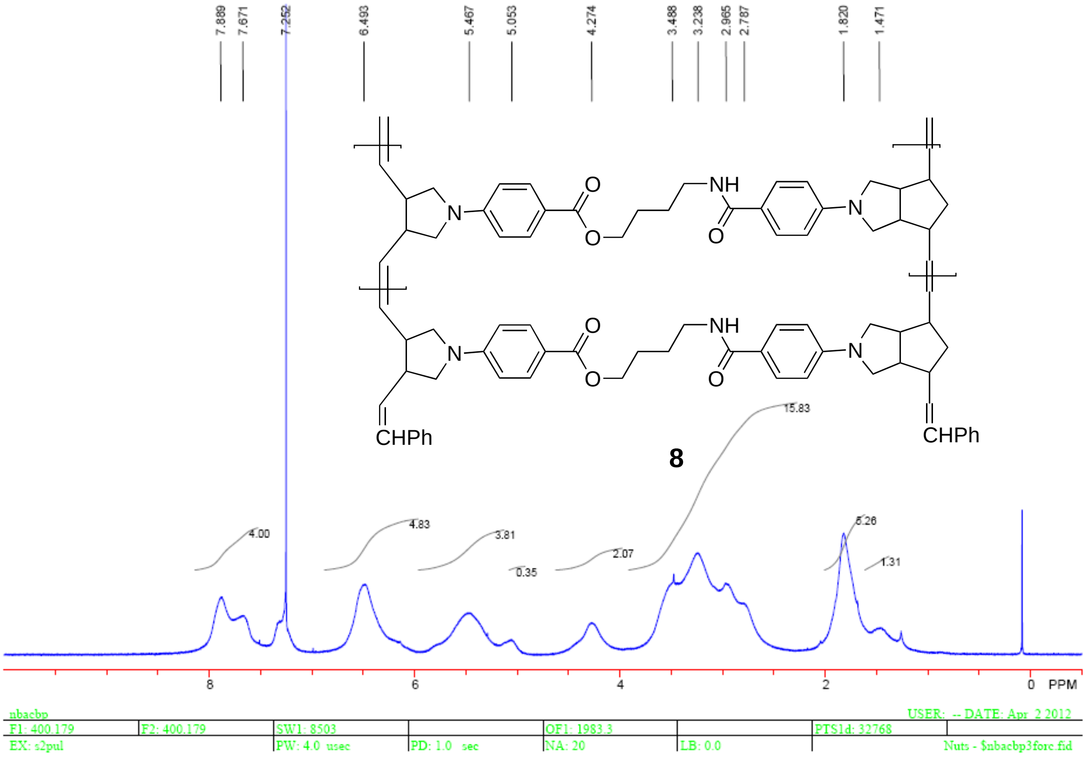

(b)

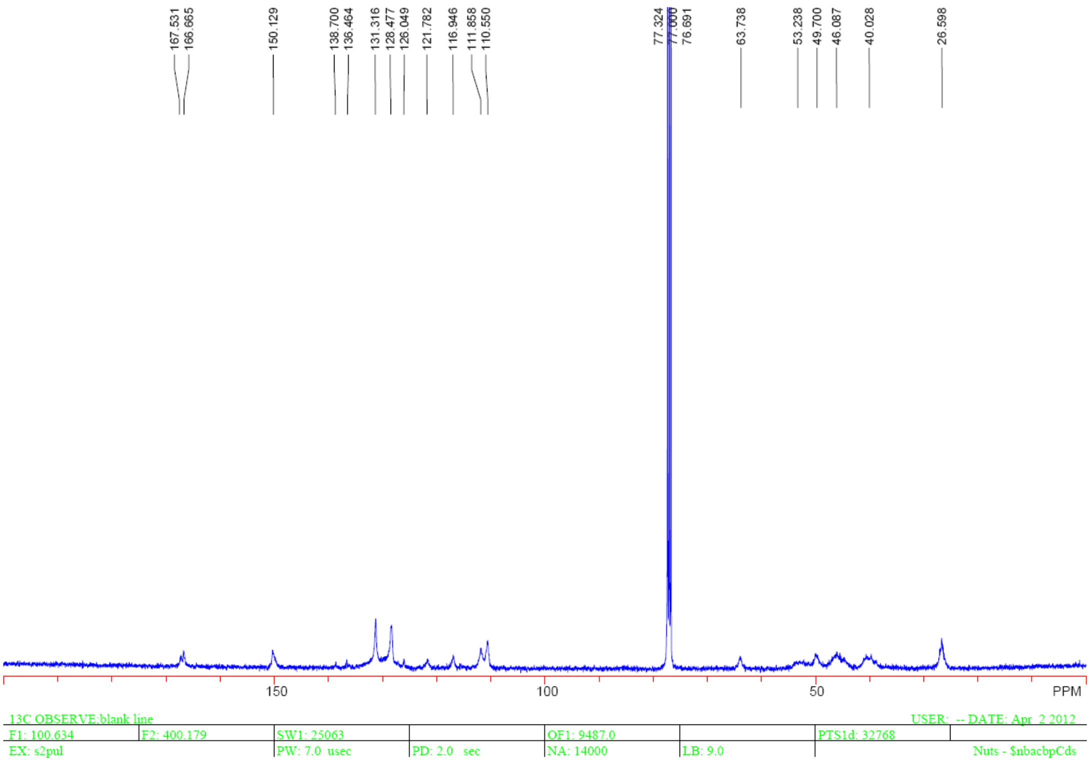

Figure S5: (a) ^{1}H (400 MHz) and (b) ^{13}C (100 MHz) NMR spectra of **8**.

(a)

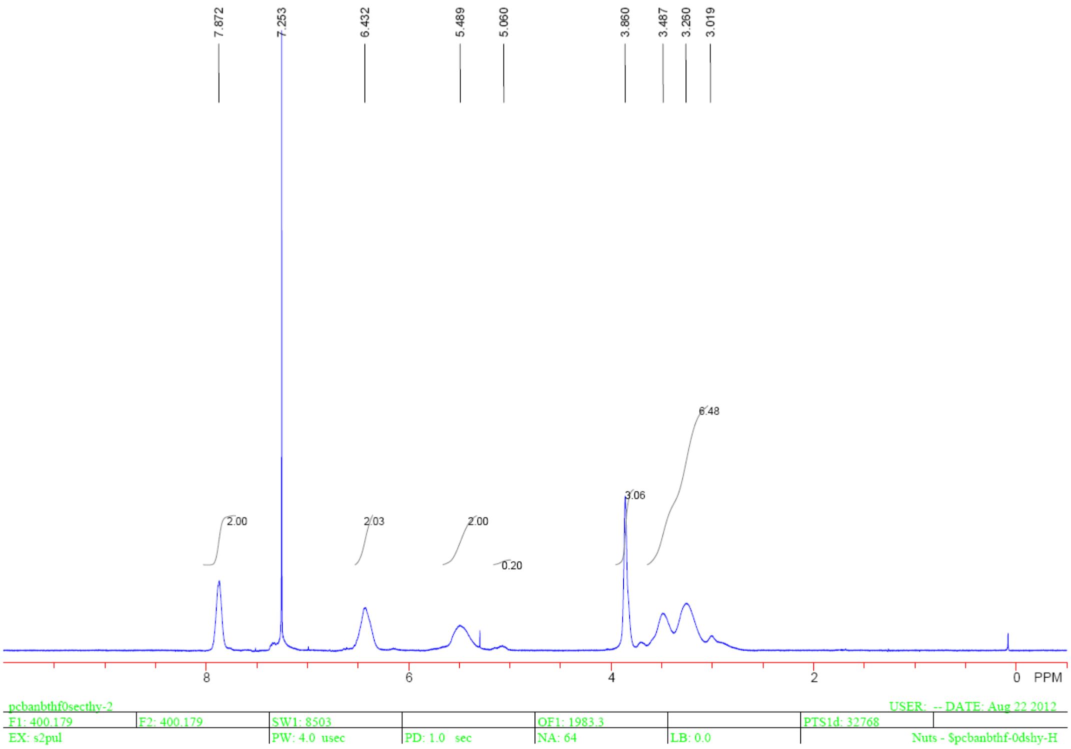

(b)

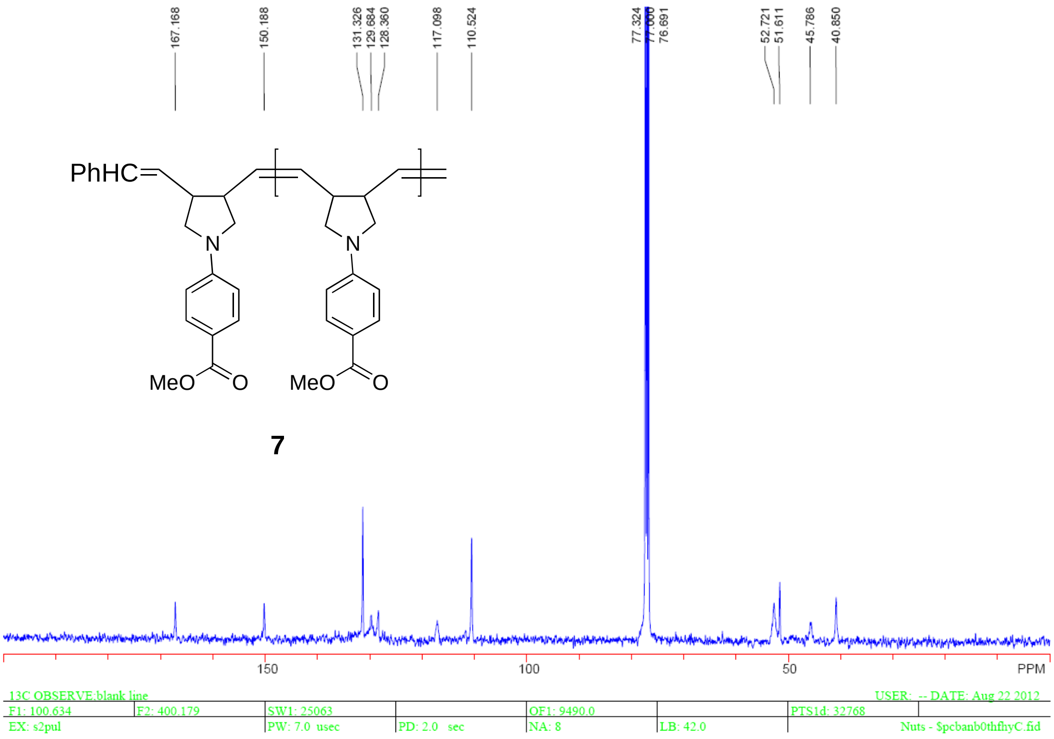

Figure S6: (a) ^{1}H (400 MHz) and (b) ^{13}C (100 MHz) NMR spectra of **7**.

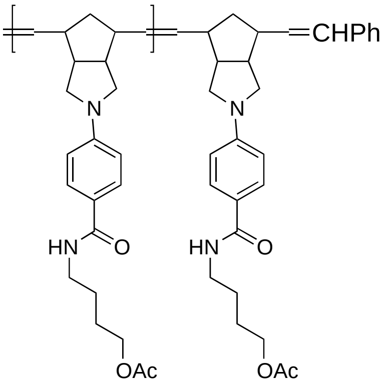

19

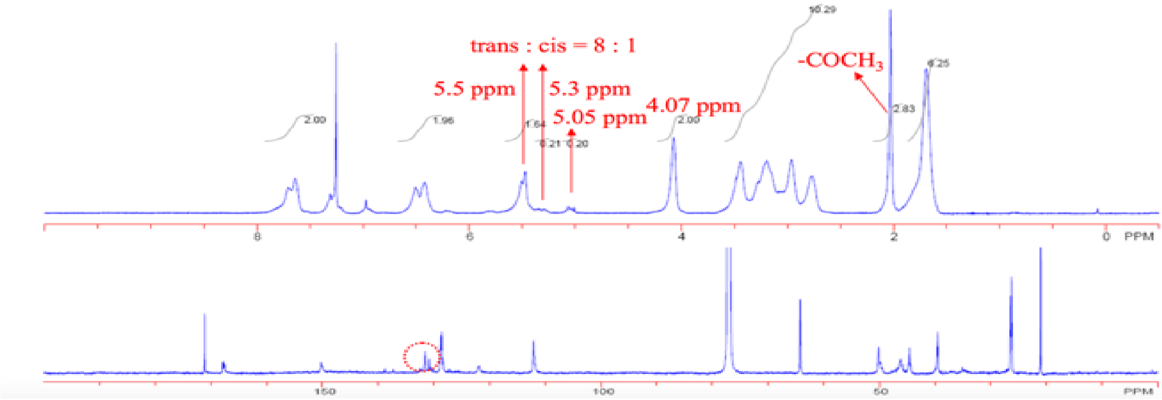

Figure S7: (a) ^{1}H (400 MHz) and (b) ^{13}C (100 MHz) NMR spectra of **19**.

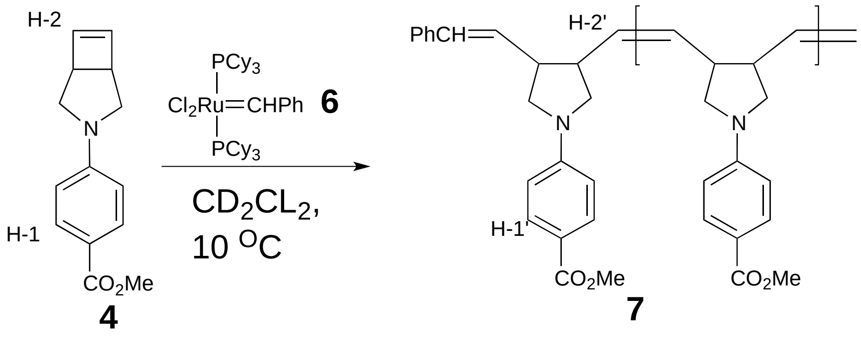

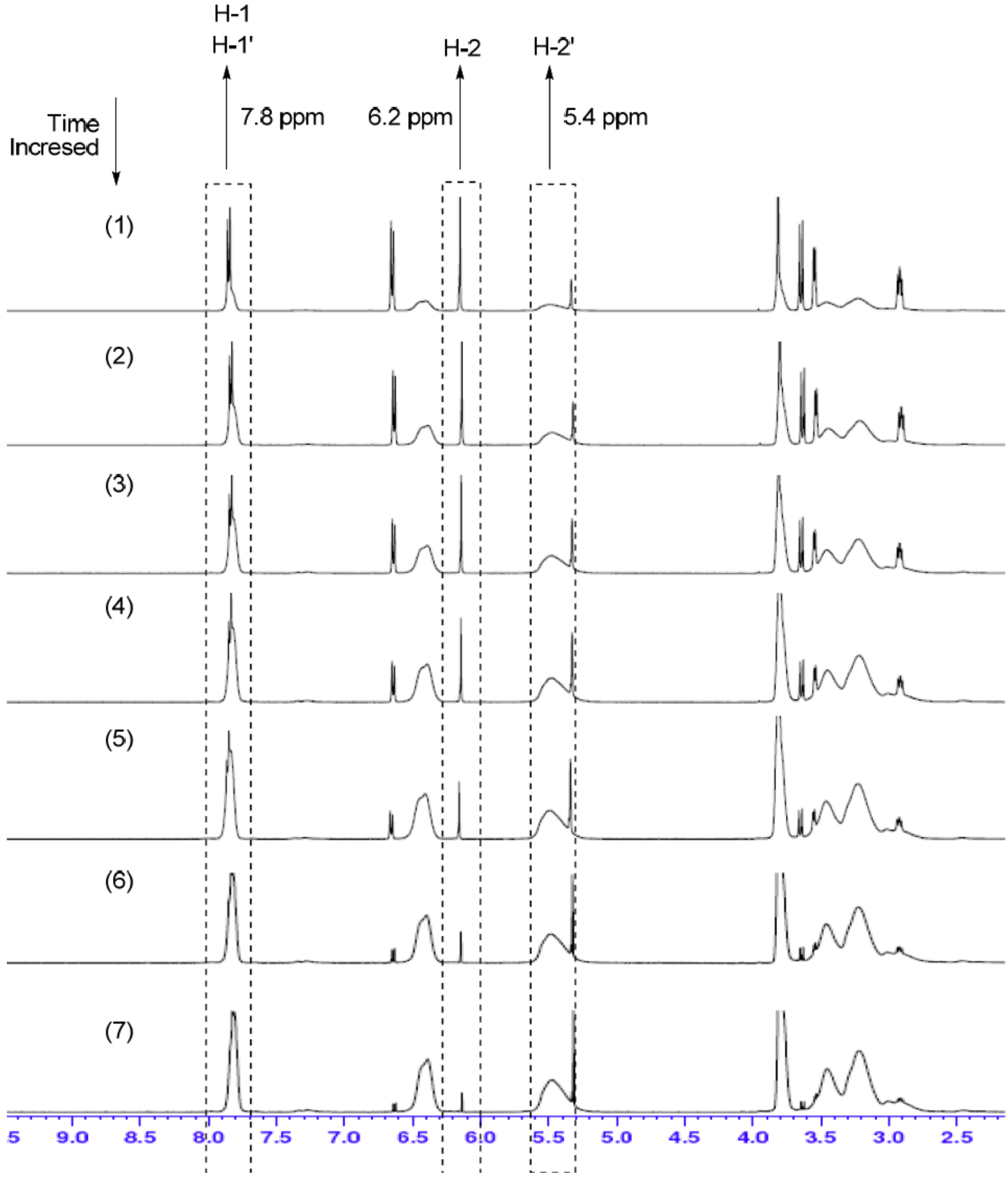

Figure S8: Kinetics of the **6**-catalyzed reaction of **4** in CD_2Cl_2 at 10 °C The reaction was monitored by the decrease of the peak intensity for H-2 using the peaks for H-1 and H-1' as the internal reference. The spectra were recorded every ten minutes.

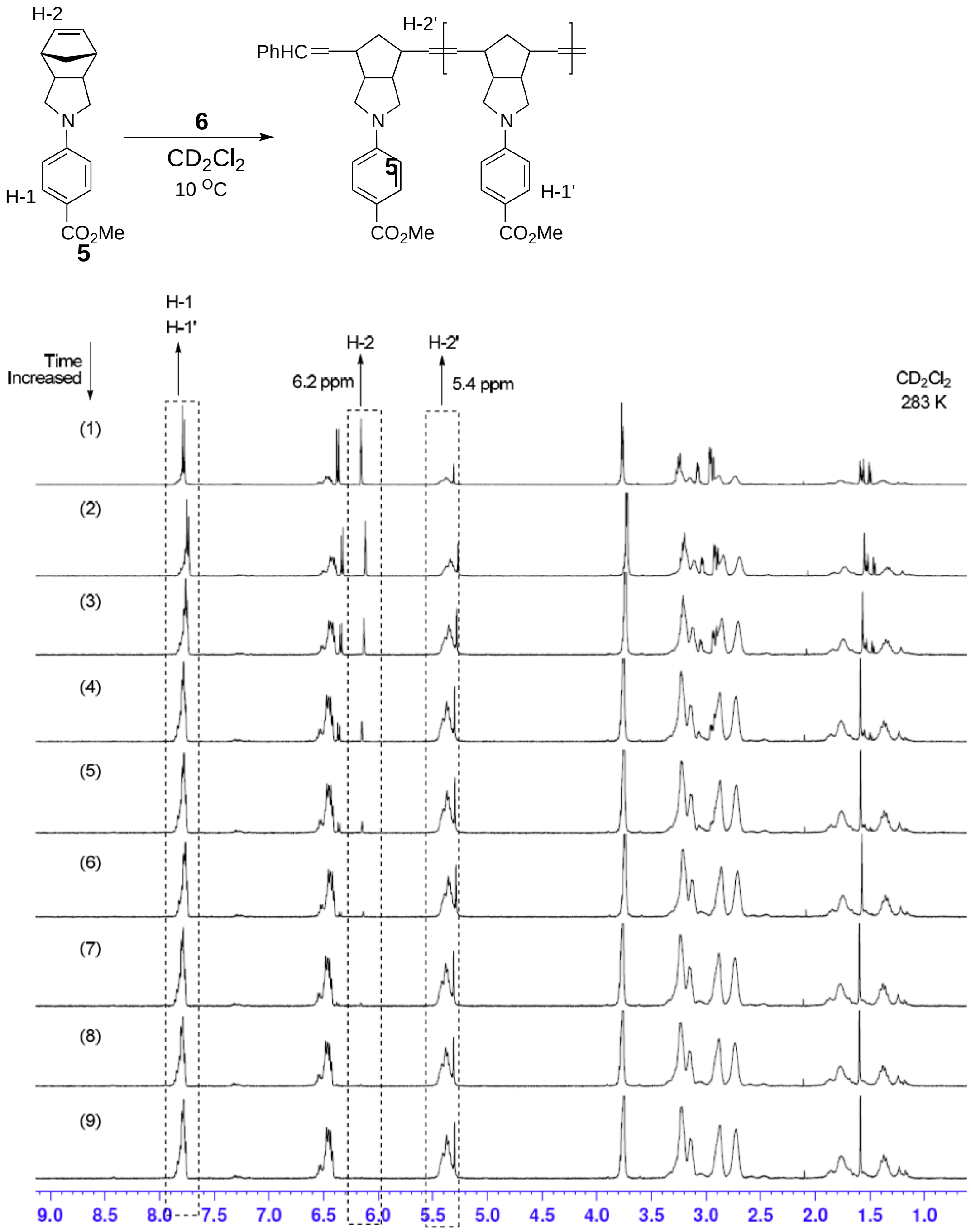

Figure S9: Kinetics of the **6**-catalyzed reaction of **5** in CD_2Cl_2 at 10 °C The reaction was monitored by the decrease of the peak intensity for H-2 using the peaks for H-1 and H-1' as the internal reference. The spectra were recorded every ten minutes.

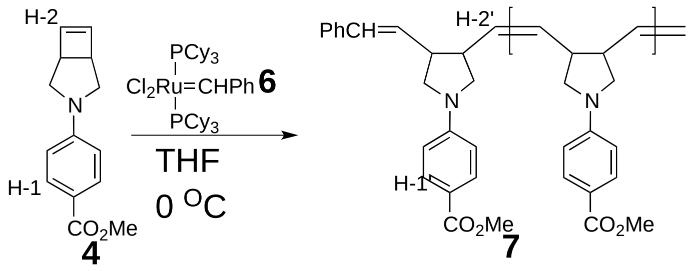

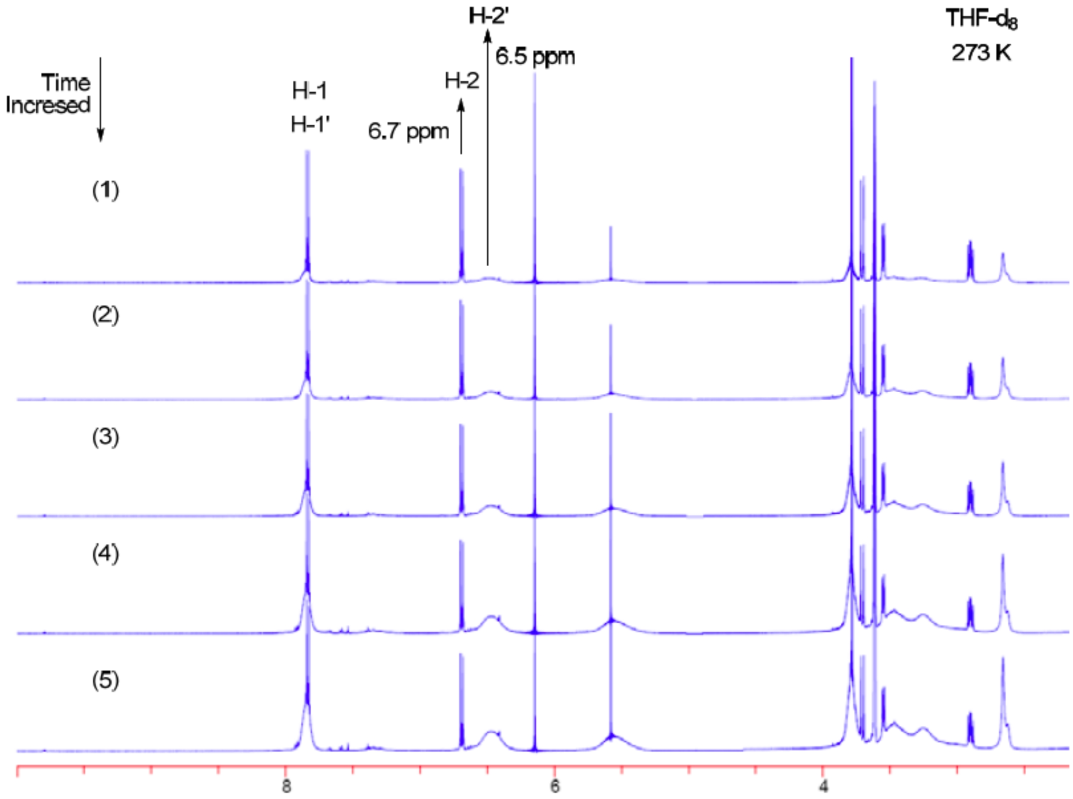

Figure S10: Kinetics of the **6**-catalyzed reaction of **4** in THF at 0 °C The reaction was monitored by the decrease of the peak intensity for H-2 using the peaks for H-1 and H-1' as the internal reference. The spectra were recorded every twenty minutes.
